# Supplementary material for: Multi‐dataset identification of innovative feature genes and molecular mechanisms in keratoconus
Source: J Cell Mol Med. 2024 Sep 19;28(18):e70079. doi: 10.1111/jcmm.70079 (PMC11412914; doi:10.1111/jcmm.70079)
Supplement: Supplementary file 2 — Material S2. [file JCMM-28-e70079-s002.zip › 7.GSEA analysis/6.SNORA74B/KEGG_BUTANOATE_METABOLISM.html]

Details for gene set KEGG\_BUTANOATE\_METABOLISM[GSEA]

|  || Dataset | SNORA74B.group.cls#h\_versus\_l |
| Phenotype | group.cls#h\_versus\_l |
| Upregulated in class | l |
| GeneSet | KEGG\_BUTANOATE\_METABOLISM |
| Enrichment Score (ES) | -0.4620255 |
| Normalized Enrichment Score (NES) | -1.3397994 |
| Nominal p-value | 0.14450867 |
| FDR q-value | 0.3124117 |
| FWER p-Value | 0.934 |
Table: GSEA Results Summary

  

Fig 1: Enrichment plot: KEGG\_BUTANOATE\_METABOLISM      
 Profile of the Running ES Score & Positions of GeneSet Members on the Rank Ordered List

  

| SYMBOL | TITLE | RANK IN GENE LIST | RANK METRIC SCORE | RUNNING ES | CORE ENRICHMENT || 1 | ACSM1 | na | 3036 | 0.299 | -0.0281 | No |
| 2 | GAD2 | na | 6760 | 0.169 | -0.1017 | No |
| 3 | HMGCL | na | 6817 | 0.168 | -0.0699 | No |
| 4 | ACSM2A | na | 8180 | 0.132 | -0.0830 | No |
| 5 | ACSM4 | na | 8385 | 0.127 | -0.0635 | No |
| 6 | ABAT | na | 10136 | 0.090 | -0.0960 | No |
| 7 | HMGCS2 | na | 10618 | 0.081 | -0.0937 | No |
| 8 | L2HGDH | na | 11735 | 0.064 | -0.1133 | No |
| 9 | AACS | na | 12162 | 0.058 | -0.1141 | No |
| 10 | ALDH3A2 | na | 13104 | 0.046 | -0.1321 | No |
| 11 | BDH1 | na | 13187 | 0.045 | -0.1255 | No |
| 12 | PDHA2 | na | 13714 | 0.039 | -0.1329 | No |
| 13 | ACSM5 | na | 15098 | 0.024 | -0.1680 | No |
| 14 | ACAT2 | na | 15497 | 0.020 | -0.1756 | No |
| 15 | HMGCS1 | na | 16538 | 0.009 | -0.2038 | No |
| 16 | ACAT1 | na | 16541 | 0.009 | -0.2021 | No |
| 17 | PDHB | na | 16643 | 0.008 | -0.2034 | No |
| 18 | ECHS1 | na | 18214 | -0.008 | -0.2470 | No |
| 19 | PDHA1 | na | 18249 | -0.009 | -0.2462 | No |
| 20 | HADHA | na | 19437 | -0.022 | -0.2760 | No |
| 21 | ALDH7A1 | na | 19848 | -0.027 | -0.2825 | No |
| 22 | ACADS | na | 19906 | -0.027 | -0.2787 | No |
| 23 | OXCT1 | na | 20622 | -0.035 | -0.2922 | No |
| 24 | HADH | na | 20699 | -0.036 | -0.2872 | No |
| 25 | ALDH9A1 | na | 25479 | -0.107 | -0.4036 | No |
| 26 | EHHADH | na | 26637 | -0.131 | -0.4109 | No |
| 27 | ALDH1B1 | na | 27039 | -0.140 | -0.3946 | No |
| 28 | GAD1 | na | 28502 | -0.180 | -0.4010 | No |
| 29 | OXCT2 | na | 30622 | -0.249 | -0.4124 | Yes |
| 30 | ALDH5A1 | na | 31138 | -0.275 | -0.3724 | Yes |
| 31 | BDH2 | na | 32151 | -0.331 | -0.3358 | Yes |
| 32 | ACSM3 | na | 34204 | -0.582 | -0.2790 | Yes |
| 33 | ALDH2 | na | 34359 | -0.636 | -0.1569 | Yes |
| 34 | AKR1B10 | na | 34632 | -0.842 | 0.0028 | Yes |
Table: GSEA details [plain text format]

  

Fig 2: KEGG\_BUTANOATE\_METABOLISM      
 Blue-Pink O' Gram in the Space of the Analyzed GeneSet

  

Fig 3: KEGG\_BUTANOATE\_METABOLISM: Random ES distribution      
 Gene set null distribution of ES for **KEGG\_BUTANOATE\_METABOLISM**
